# The Influence of Pore-Forming Diluents on Porous Structure, Thermal and Sorption Properties of the Divinylbenzene and Glycidyl Methacrylate Copolymers

**DOI:** 10.3390/ma17164114

**Published:** 2024-08-20

**Authors:** Magdalena Sobiesiak, Monika Parcheta

**Affiliations:** Department of Polymer Chemistry, Faculty of Chemistry, Maria Curie-Sklodowska University, pl. Marii Curie-Sklodowskiej 5, 20-031 Lublin, Poland

**Keywords:** polymeric microspheres, porous structure formation, methylene blue sorption, TGA, divinylbenzene, glycidyl methacrylate, copolymers

## Abstract

The aim of this work was the characterization of polymer microspheres obtained by the suspension polymerization of divinylbenzene (DVB) and glycidyl methacrylate (GMA), depending on the pore-forming diluents and molar ratio of monomers. The assessed properties included the chemical and porous structure, thermal stability, and sorption capacity of the obtained polymers towards methylene blue. The abovementioned characteristic was carried out for two series of copolymers with molar ratios of monomers of 1:2, 1:1 and 2:1, synthetized with toluene and a mixture of decanol and benzyl alcohol. The structure of the polymers was confirmed by FTIR and elemental analysis. The results of TGA demonstrated the main influence on thermal stability was the composition of polymers, whereas the impact of porogens was negligible. The S_BET_ varied in the range of 12–534 m^2^g^−1^ for polymers obtained with toluene and 0–396 m^2^g^−1^ with the mixture of alcohols. Toluene enhanced the formation of micro- and mesopores, while the mixture of alcohols enhanced the creation of meso- and macropores. For the polymers prepared with toluene, their effectiveness in water purification decreases in the following order: DVB-GMA 2:1 > DVB-GMA 1:1 > DVB-GMA 1:2, according to the decreasing values of porous structure parameters. In the case of a series obtained with a mixture of alcohols, such correlation was not observed.

## 1. Introduction

Progressive development and industrialization significantly affect the natural environment, causing pollution with toxic chemical substances, such as medicines, artificial dyes, heavy metals, and chemical warfare agents. The spread of these compounds in the environment contributes to their incorporation into the animal food chain, and thus has an inextricable impact on human health [1]. Artificial dyes entering the aquatic environment along with sewage from the textile industry limit the light penetration, which results in the impaired photosynthetic activity of aquatic plants. One of the most serious threats is the textile industry, responsible for releasing an average of 20,000 tons of artificial dyes into the natural environment per year, accounting for 17–20% of the global water pollution [2,3]. The dyes most commonly used in the textile industry are chemical compounds containing one or more azo groups in their structure. After entering the human body, azo dyes are transformed by the intestinal microflora into toxic intermediates, i.e., carcinogenic aromatic amines [4]. The toxicity of these amines results from their metabolic oxidation to diazonium salts, which, as electrophilic reducing intermediates, have the ability to form a covalent bond with DNA [5].

Due to its wide availability and low price, one of the dyes commonly used for coloring silk, cotton and wool fabrics, but also in the paper and tanning industry, is methylene blue, which belongs to the group of thioazo dyes [6]. As a result of metabolic processes, methylene blue is transformed into carcinogenic methylene amines and benzidine. Studies conducted on microalgae, i.e., *Spirulina platensis* and *Chlorella vulgaris*, have shown that the increasing concentration of this pigment leads to the limited growth of these organisms and also causes a decrease in the concentration of chlorophyll in their cells, significantly contributing to limiting their development and photosynthetic abilities [7].

In the pharmaceutical industry, methylene blue is used as a drug against Alzheimer’s disease [8], Malaria, and in the treatment of fungal infections of the skin, oral cavity and esophagus, digestive system, vagina, and vascular system in humans, caused by *Candida* yeasts [9]. Another dangerous metabolite of methylene blue is a cationic dye—Azure B—which also can be incorporated into the structure of DNA and RNA. This metabolite is able to inhibit glutathione reductase in the human body, which is a key enzyme in maintaining cellular redox balance [10].

Therefore, it is extremely important to remove dyes such as methylene blue from the aquatic environment as effectively as possible. For this purpose, biological techniques such as biodegradation, chemical techniques such as flocculation, using metal ions as substances to induce this process, and physical techniques such as filtration, reverse osmosis, adsorption, or electrolysis are used. Due to its ability to handle high flow rates, low process costs, and high efficiency, adsorption is considered the most versatile water treatment process, especially in developing countries [11]. The possibility of controlling the pore size and chemical and physicochemical properties makes polymer adsorbents of increasing scientific interest [12].

In this publication, we propose polymer microspheres as an adsorbent for water treatment. Microspheres are particles ranging in size from 1 to 1000 µm, characterized by a high surface area to volume ratio. Polymer adsorbents can take various forms, i.e., macroporous, hollow, nanofibers or core–shell [13]. The most frequently used methods for preparing polymer microspheres include emulsion or suspension polymerization, swelling polymerization, controlled polymerization, and precipitation polymerization. Since many factors influence the morphology of microspheres, one of the greatest challenges encountered during the synthesis of microspheres is preventing discrepancies in their shape and particle and pore size [14].

Currently, polymer microspheres are successfully used as sorbents, carriers for controlled drug release, fillings for chromatographic columns, microreactors in heterogeneous catalysis and as biomedical materials. Very often, in order to adapt polymer microspheres to their intended use, e.g., as chelating materials or ion exchangers, chemical modifications of their surface are desired. Such modifications allow for the introduction of highly reactive functional groups, i.e., aldehyde, hydroxyl, amine or thiol groups, into the polymer surface [15]. Due to the precisely defined structure, polymer microspheres meet the requirements for column fillers for chromatographic analyses and the solid phase extraction (SPE) technique. Good polymer sorbents should be characterized by high sorption capacity, significant stability, high regenerative capacity, and low production costs [16]. Another aspect that cannot be overestimated is their thermal stability. The thermal properties of cross-linked polymers determine their suitability in many different techniques requiring temperatures often exceeding 200 degrees Celsius, i.e., gas chromatography or high-temperature catalytic processes [17].

**Figure 1:**
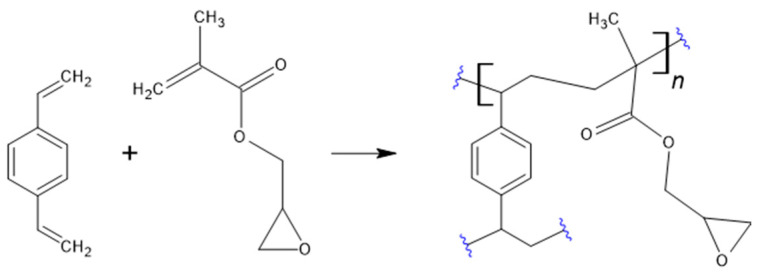
Polymerization of DVB and GMA 1:1 [18].

In this work, a comparison of the thermal, porous, and sorption properties of copolymers based on divinylbenzene (DVB) and glycidyl methacrylate (GMA), prepared with two different pore-forming systems, is presented. Figure 1 shows the hypothetical structure of the polymer with a monomer ratio of 1:1. All the polymers were synthesized using suspension polymerization to obtain materials in the form of microspheres. To create porosity, toluene and mixture of decanol and benzyl alcohol were applied.

## 2. Materials and Methods

### 2.1. Materials

Polyvinyl alcohol 72,000, hydrolyzed in 85–89% (PVA) and toluene (t) were purchased from POCH (Gliwice, Poland); decanol (d), α,α’-asoiso-bis-butrylonitryle (AIBN), benzyl alcohol (b), divinylbenzene (DVB) and glycidyl methacrylate (GMA), methanol were bought from Sigma Aldrich (Stenheim, Germany), and methylene blue (MB) from POCH (Gliwice, Poland).

### 2.2. Synthesis of Polymeric Microspheres

DVB homopolymer and DVB and GMA copolymers were synthesized in a three-necked flask equipped with a stirrer, a thermometer and a reflux condenser. Polyvinyl alcohol solution was used as a dispersion medium (12.5 g PVA in 500 cm^3^ distilled water). The synthesis of DVB homopolymer and its copolymers with GMA in molar ratios of 1:1, 1:2 and 2:, respectively, was carried out in two different systems of pore-forming diluents: toluene (t) and a mixture of decanol and benzyl alcohol (db). The details of the synthesis parameters are listed in Table 1. The mixture of monomers and pore-forming diluents with dissolved AIBN (0.15 g, 0.5% of the monomers’ mass) as the reaction initiator were poured to the dispersion medium. The synthesis was carried out for 20 h at 80 °C. The reaction mixture was stirred at 300 rpm. The obtained product was filtered off using a Buchner funnel and washed with hot water. Next, the polymeric microspheres were transferred to a Soxhlet apparatus, where they were washed with methanol for at least 10 cycles. The synthetized polymers had a broad microsphere size distribution; therefore, it was divided into sieve fractions in the range of 0–1.25 mm, and for the further studies a 0–0.16 fraction was used.

### 2.3. Measurements

The structure of the obtained polymers was confirmed by spectroscopic methods, using the ATR FTIR technique, using the BRUKER FTIR spectrophotometer TENSOR 27 with a resolution of 4 cm^−1^. The measurements were carried out in the frequency range of 4000–600 cm^−1^.

Elemental analyses (CHN) were performed using a Perkin Elmer CHN 2400 analyzer (Palo Alto, CA, USA).

TG/DTG curves were determined using a STA 449 F1 Jupiter thermal analyzer (Netzsch, Selb, Germany). Measurements were made in alumina crucibles, under a helium or synthetic air atmosphere, with a gas flow of 30 mL min^−1^. Dynamic scans were carried out in the temperature range of 20–1000 °C, and the heating rate was 10 °C min^−1^. The sample weight was about 7 mg and the measurements were made in the presence of an empty crucible as a reference.

Images of polymeric microspheres were taken with optical microscope (Malvern Morphologi G3 with, Morphologi G3 software). The magnification was set on 10×.

The porous structure of the studied polymers was investigated using the ASAP 2405 adsorption analyzer (Micromeritics Inc., Norcross, GA, USA). Nitrogen adsorption–desorption measurements were performed at −190 °C, after degassing the samples at 70 °C. The specific surface area was calculated by the standard BET method (S_BET_), while the total pore volume (V_tot_) was evaluated as the volume of the liquid adsorbed at 0.99 relative pressure. The pore size distribution (PSD) was calculated from the adsorption branch of the isotherm using the 2D-NLDFT model, available as free version from the SAIEUS 3.0 software (Micromeritics) on www.nldft.com.

The efficiency of removing methylene blue from an aqueous solution was performed using a dynamic sorption method. For this purpose, SPE columns with a diameter of 1 cm and a height of 5 cm were used. The columns were filled with a 100 mg of the tested polymer placed between two Teflon disks. The columns were conditioned with 2.5 cm^3^ of methanol and then 5 cm^3^ of distilled water. Sorption studies were carried out at room temperature. An aqueous solutions of methylene blue (MB) of stock concentration equal to 1 mg/L were passed through the bed. The eluate was collected in separate fractions with a volume of 10 cm^3^. The MB content in each fraction was determined photometrically using a UV-1800 spectrophotometer from Schimadzu (Canby, OR, USA). Changes in the concentration of methylene blue were determined on the basis of a calibration curve (λ = 666 nm). An increment of the MB concentration in each fraction expressed as a dependency C/C_0_ = f(V) was used to plot the breakthrough curves.

The sorption capacity *q_a_* [mg/g] was calculated on the basis of Equation (1), where *C*_0_ is the concentration of stock solution, *C_i_* is the concentration of the solution leaving the column, *V_i_* is the volume [L] of a single fraction of methylene blue solution.

(1) $$q_{a}=\frac{\sum (C_{0}-C_{i})}{m},$$

Total mass adsorbed was calculated as a percentage value of mass adsorbed until the total breakthrough of the column in relation to the mass introduced into the column, as is shown in Equation (2). All symbols as above; *n*—the number of fractions when breakthrough volume was reached.

(2) $$Total\;mass\;adsorbed=\frac{\sum_{i=1}^{n}(C_{0}-C_{i})\cdot V_{i}}{\sum_{i=1}^{n}C_{0}\cdot V_{i}}\cdot 100\%,$$

In order to assess the reversibility of the MB adsorption, a desorption test was carried out using methanol, which has a high elution strength (0.95) [19]. The polymers with adsorbed MB were treated with 3–5 portions of methanol with a volume of 3 mL for each portion (*V_des i_*), until the eluate was clear. The concentration of MB in methanolic eluates (*C_des i_*) was determined in the same way as in case of the aqueous ones, using a calibration curve for methanolic solutions of MB. Desorbed mass of methylene blue was expressed as the percentage value of MB mass retained on the polymer in relation to the total mass adsorbed and designated according to Equation (3).

(3) $$Mass\;desorbed=\frac{\sum_{i=1}^{n}(C_{des\;i}\cdot {Vdes\;}_{i})\cdot 100\%}{Total\;mass\;adsorbed}$$

The mass retained on the bed was calculated as a difference between the total mass adsorbed and the mass desorbed.

## 3. Results and Discussion

### 3.1. Chemical Structure Characterization

**Figure 2:**
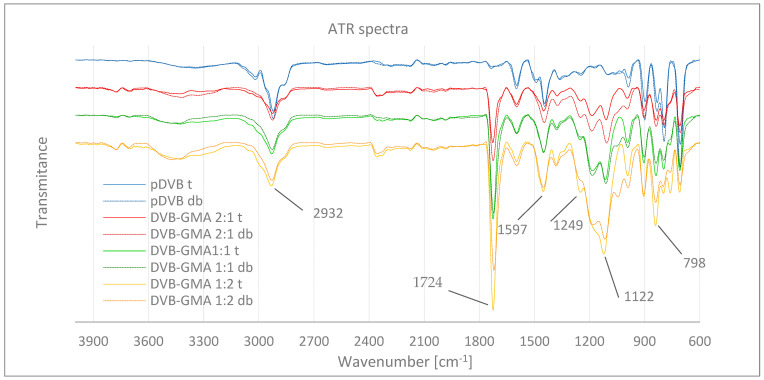
The ATR FTIR spectra of synthesized polymers for both applied pore-forming systems: toluene (t) and a mixture of benzyl alcohol and decanol (db).

The structure of the obtained polymers was confirmed by the ATR FTIR technique. The collected spectra are shown in Figure 2. To visualize the differences better, the spectra were grouped into pairs with the same chemical composition but different porogenic systems (t-toluene, db-mixture of decanol and benzyl alcohol), and stacked by different material compositions.

The positions of the characteristic FTIR bands of the polymers structural elements are presented in Table 2.

For the pDVB polymers prepared with the use of toluene or the alcohol mixture, no significant differences can be observed. The superimposed plots overlap with good accuracy. The most intense bands in the fingerprint range are due to out-of-plane ring deformation vibrations (γ(ArH) and γ(Ar) at about 706, 794, 831 and 900 cm^−1^). Above this region, medium intensity bands are caused by stretching vibrations of the aromatic system (ν_s_ and ν_as_ from 1625 to 1430 and ν(ArH) 3100 to 3000 cm^−1^) and aliphatic C-H bonds (ν_s_ and ν_as_ from 3000 to 2800 cm^−1^). The only difference concerns the relative ratio of two bands intensities, that at 900 and 830 cm^−1^. In the case of the pDVB t, their intensities are the same, while in pDVB db the former is more intense than the latter. The wavenumber of 900 cm^−1^ can also be assigned to =CH_2_ out-of-plane deformation vibration in vinyl group. This observation leads to the conclusion that in the structure of pDVB db there are still unreacted vinyl bonds. The more detailed interpretation of the pDVB ATR spectrum can be found in our previous work [21].

In the spectra of DVB-GMA copolymers, characteristic bands of GMA are visible [18]. Their intensities increase as the content of the functional monomer increases. Among them, vibrations of ester and epoxide groups are the most indicative. The formers are located at about 1726 (ν(C=O)) and in the range of 1200–1100 cm^−1^ (ν_s_ and ν_as_ (C-O)), with the latter at 1249 (ν_s_ (C-O of epoxide ring)), 905 and 836 cm^−1^ (δ_s_ and δ_as_ (epoxide ring)). Despite the chemical similarity of these materials, some differences can also be noted. They are the most pronounced for the polymers where GMA predominates (DVB-GMA 1:2) and concern the presence or absence of some bands or changes in their intensities, especially in the range of 1500 to 950 cm^−1^. Comparing the spectra of DVB-GMA 2:1 samples, one can see that for DVB-GMA 2:1 db bands of the GMA have stronger intensities, while those from DVB are the same as in DVB-GMA 2:1 t. This indicate the polar solvents system (alcohols) increases the retention of GMA in the organic phase of the reaction mixture; therefore, a larger amount of GMA monomer can be incorporated into the polymer network. In the case of DVB-GMA 2:1 t, where toluene is used as the porogene, the solubility of GMA is limited, which results in its slightly smaller share in the polymer structure. These results are also in good agreement with the elemental analysis data (Table 3). On the other hand, toluene is less reactive compared to the alcohols in the porogenic mixture. For this reason, in the spectra of the DVB-GMA t copolymer, all epoxide bands can be easily identified (ca. 1249, 900, 840, 800 and 762 cm^−1^). However, not all of these bands are present in the spectra of the DVB-GMA 2:1 db polymer. For instance, vibrations at 1249 and 762 cm^−1^ practically disappeared, while the rest decreased (significantly). These changes are accompanied by the appearance of a new band at ca. 1050 cm^−1^ (ν(O-H)) and an intensification in the range 3200–3600 cm^−1^, suggesting the partial decomposition of the epoxide ring with the formation of a primary alcohol and an ether (bands in the same range as for esters) as substitution products. The mechanism of an epoxide ring opening reaction was already described in a previous paper [18]. This is probably why the originally well-separated bands originating from C-O-C vibrations (ν_s_ and ν_as_) merged into one with a shoulder on the higher wavenumbers side.

The registered infrared spectra confirm an intended course of the syntheses, especially the presence of peaks coming from vibrations of ester bonds in the range of 1200–1100 cm^−1^ and at 1724–1726 cm^−1^ for toluene and 1720–1724 cm^−1^ for decanol and benzyl alcohol as a pore-forming diluent, respectively. The intensity of these vibrations increases with the increasing volume fraction of GMA in the polymer. The spectra also show bands from stretching vibrations of the aromatic ring at 708–710 cm^−1^ and from deformation vibrations of the ring at 1109 cm^−1^ and 1107 cm^−1^ for polymers obtained, respectively, in the presence of the above-mentioned pore-forming diluents. The spectra of the tested polymers also show low-intensity peaks coming from the vibrations of the vinyl group in the range of 998–901 cm^−1^. Their presence may indicate the existence of unreacted unsaturated bonds in the polymer structure. The low intensities of these bands prove a high degree of cross-linking of the prepared polymer microspheres [18].

The results of the elemental analysis collected in Table 3 allow us to compare the chemical composition of the synthetized polymers with the theoretical values calculated based on the assumed molar ratio. All the determined results are very close to the theoretical values, confirming the expected course of the reaction. The best correlation between experimental and theoretical values was observed for DVB-GMA 1:1 polymers. Quite good similarity was also obtained for pDVBs; however, a content of oxygen of approximately 2% indicates the slight oxidation of the polymer. The presence of such oxidized moieties on the polymeric surface can be verified by FTIR spectra, which show very weak characteristic bands of C=O and H-O groups at about 1700 and 3200–3600 cm^−1^, respectively.

The greatest difference of up to about 4% was found for the copolymer DVB-GMA 2:1 db. It should be noted that in the case of this copolymer, the carbon content is lower and the oxygen content is higher than theoretically assumed. The observed phenomenon is in line with the above description, explaining some differences in the FTIR spectra of the DVB-GMA 2:1 t and DVB-GMA 2:1 db samples.

### 3.2. Thermal Properties of the Studied Polymers

TGA allows us to evaluate thermal stability of the synthesized polymers. The analyses performed under helium atmosphere enable us to study the thermal degradation process and can be very useful when polymers are applied in an elevated temperature (e.g., gas chromatography, thermal desorption). The analyses carried out in a synthetic air atmosphere let us investigate the thermal behavior of the materials under oxidizing conditions, when thermal decomposition is accompanied by a reaction with oxygen.

**Figure 3:**
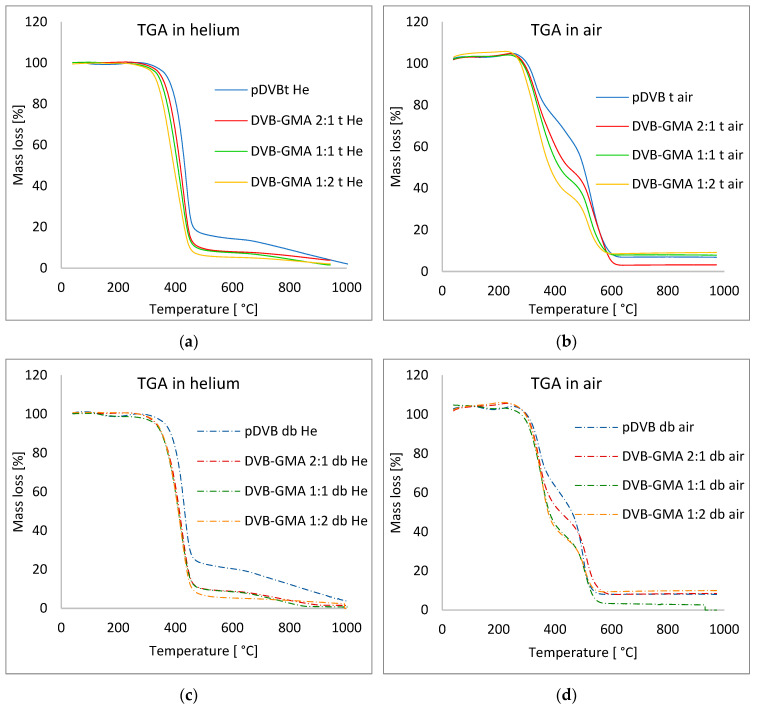
The influence of the applied pore-forming diluents on the thermal behavior of the analyzed polymers under a helium and air atmosphere. TG curves of the toluene series in He (**a**) and in the air (**b**), and the mixture of alcohols series in He (**c**) and in the air (**d**).

Figure 3 presents the TGA curves of the prepared polymers under both He and synthetic air, and some characteristic parameters are collected in Table 4.

For the series of the polymers prepared with toluene (Figure 3a,b), a relationship between chemical composition and thermal stability is evident. As the GMA content in the polymer structure increases, the decomposition process occurs at a lower temperature. This tendency is a consequence not only of the presence of the GMA monomer in the copolymer, but also of the lower cross-linking density of the network [22,23,24,25,26,27,28]. A similar trend can be observed for the series synthesized with mixture of alcohols; however, in this case, differences are smaller.

**Figure 4:**
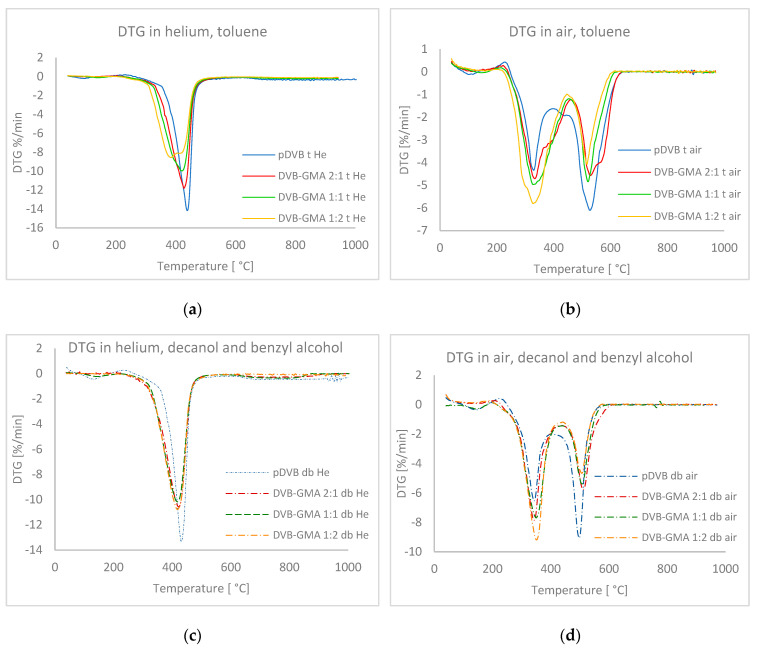
DTG curves recorded under helium and synthetic air conditions. DTG curves of the toluene series in He (**a**) and in the air (**b**), and the mixture of alcohols series in He (**c**) and in the air (**d**).

A comparison of the data collected for both series under the He atmosphere shows that although decomposition occurs in the same temperature ranges (T_range_ and T_max_) for polymers with the same composition, the course of the processes varies. The decomposition rate values (DR in Table 4 and Figure 4a,c) for DVB-GMA t copolymers decrease, while for DVB-GMA db they remain almost unchanged and amount to approximately 10 °C/min.

Some differences are also visible for the analyses performed under synthetic air. Initial decomposition temperatures (T_2%_) and T_range_ values gradually decrease, with nearly constant T_max_ (~330 and ~520 °C) and DR in the range of −4 to −6 °C min^−1^ for both main decomposition steps in case of the series prepared with toluene. The DTG curves presented in Figure 4b clearly indicate that although two stages of a relatively mild course have been distinguished, the process is much more complex. In all DTG plots of materials prepared with the mixture of decanol and benzyl alcohol as porogens, only two stages can be perceived. Their rates are higher than those for the corresponding polymers in the series with toluene, indicating the more rapid course of their decomposition [23].

**Figure A1:**
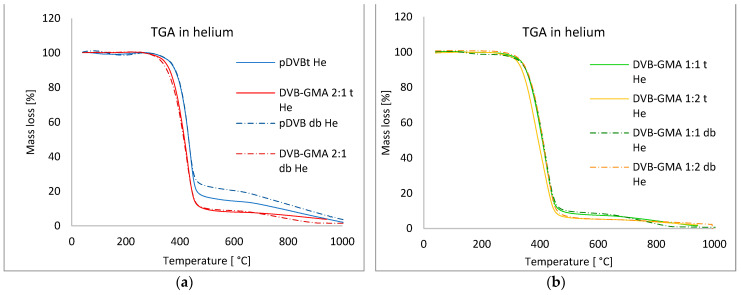

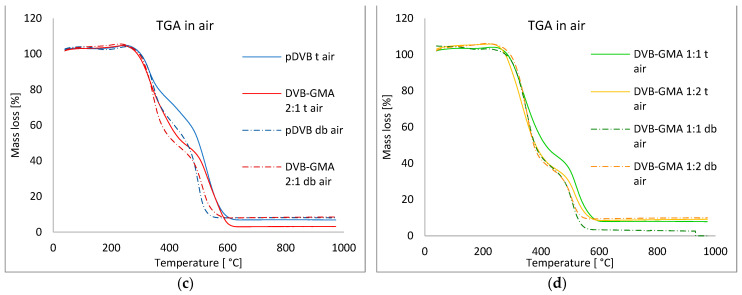
Comparison of the thermal behavior of the polymers with the same chemical composition prepared with different pore-forming agents. TGA of pDVB and DVB GMA2:1 in He (**a**), TGA of DVB-GMA 1:1 and DVB GMA1:2 in He (**b**) TGA of pDVB and DVB GMA2:1 in the air (**c**), TGA of DVB-GMA 1:1 and DVB GMA1:2 in the air (**d**).

In order to evaluate if the applied pore-forming diluents had any influence on the thermal stability-prepared materials, the TG curves of the polymers with the same monomer ratios were in pairs superimposed on the same graph (Figure A1).

In the range of polymer decomposition temperatures for analyses carried out under He (Figure A1a,b), no significant differences can be noticed, except the one for the DVB-GMA 1:2 copolymers, of which this obtained with toluene turned out to be slightly less thermally stable than that with the mixture of alcohols. Under the air atmosphere (Figure A1c,d) in terms of weight loss up to approximately 20%, no differences were recorded, with the exception of DVB-GMA t and DVB-GMA db as before. Above this value, the polymers of the db series decomposed easier than those prepared in toluene.

The performed analyses show that the chemical composition of polymers and the degree of their cross-linking determine their thermal properties to the greatest extent. The impact of the used pore-forming solvents seems to be insignificant, especially since the observed differences concern the range of temperatures in which the decomposition of the polymer under the air atmosphere is advanced (20% of the samples decomposed).

### 3.3. Morphology of Studied Polymers

**Figure 5:**
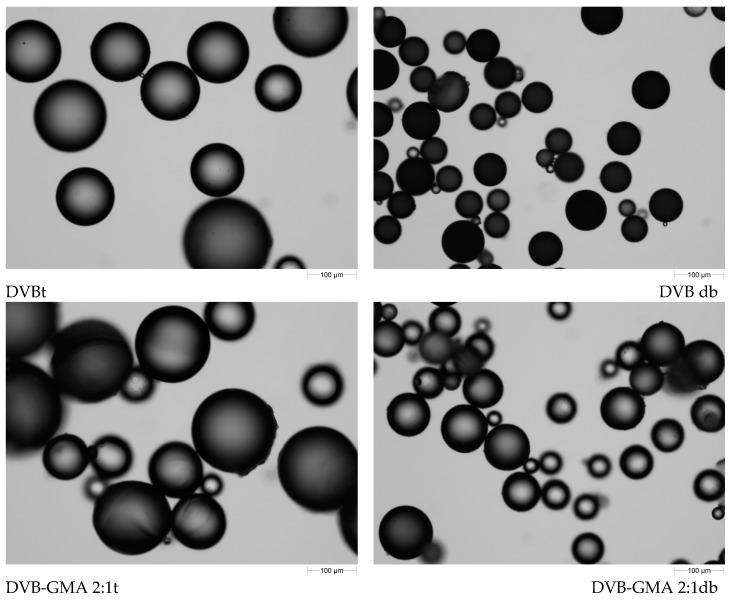

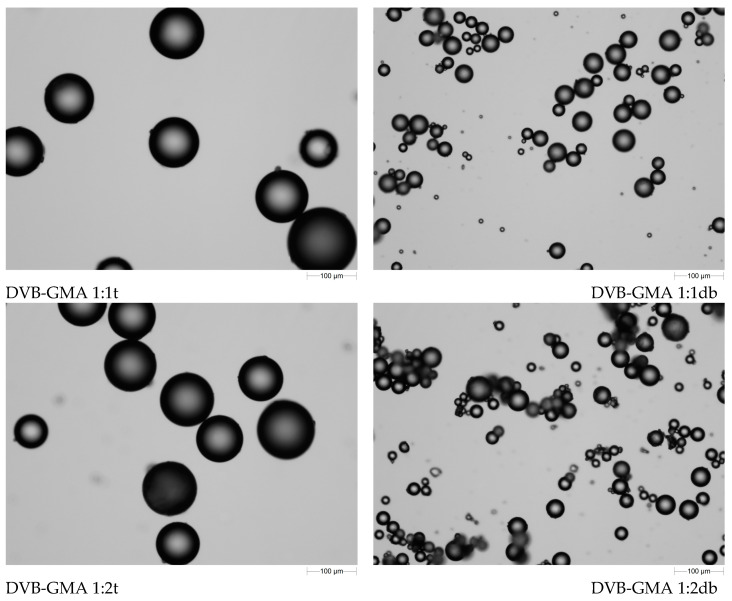
Images of the synthesized polymers taken using an optical microscope. Magnification 10×.

One of the methods for assessing the shape and size of microspheres is optical microscopy. Images taken for the studied materials are shown in Figure 5. The left column of the figure contains the photos of polymers prepared with toluene as a pore-forming agent, while those in right column are for the series prepared with the mixture of alcohols.

All synthesized materials possess regular spherical shapes. In the series prepared with toluene, large particles dominate, but their size decreases with increasing GMA content in the polymers. Most of the pDVB t microbeads have diameters of 100–160 µm, while in the case of DVB-GMA 1:2 t, the largest ones have diameters of only 100 µm. Polymers synthesized using mixture of decanol and benzyl alcohols are in all cases smaller than those in previous series. Even pDVB db diameters do not exceed 100 µm. The subsequent materials containing higher GMA content are increasingly finer. The largest particles of DVB-GMA 1:1 db and DVB-GMA 1:2 db are ca. 50 µm in diameter. In the latter material, conglomerates are formed by particles agglomerating together. Taking into account that all the polymers were prepared according to the same procedure, it can be concluded that both the chemical composition of the polymers and the used pore-forming solvents influence the size of resulting particles.

The porous structure of the prepared polymers was determined by the low temperature nitrogen adsorption–desorption method. The obtained results of the measurements are presented in Table 5.

The largest specific areas and the greatest values of total pore volumes were received for pDVB, while the poorest development of porosity was observed for the DVB-GMA 1:2 copolymers possessing the least contents of crosslinking monomer (DVB). This finding is in good agreement with the literature reports [20,21,22]. DVB, with two vinyl groups, is able to form connections between adjacent polymer chains. As its amount in the polymer compositions increases, the cross-linking density of the polymeric network increases, which results in the better development of porosity. Consequently, a decrease in specific surface area can be observed in both tested series with a decrease in the cross-linking monomer content. Additionally, an influence of porogen solvents is also visible. Larger S_BET_ were obtained for series with toluene. In the case of the DVB-GMA db series, fewer cross-linked copolymers (1:1 and 1:2) were nonporous.

**Figure 6:**
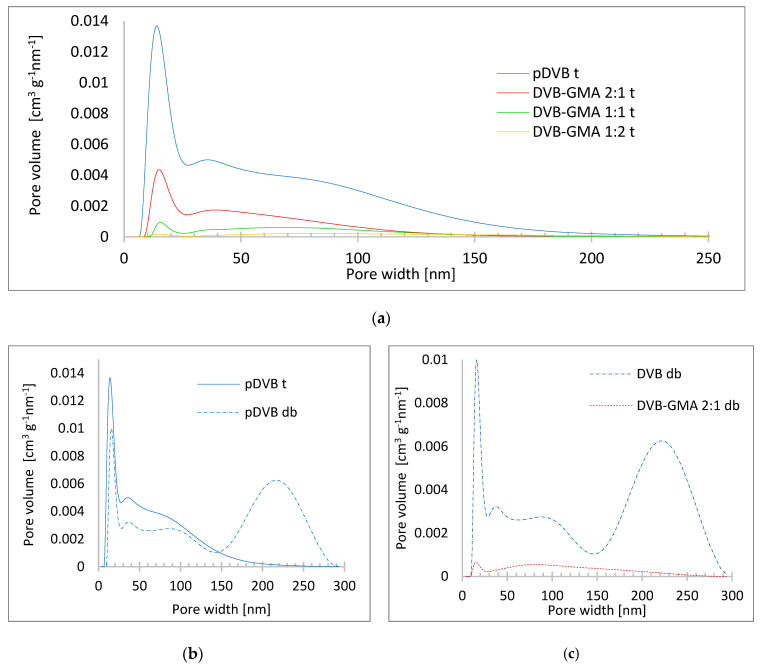
Pore size distribution curves of the obtained polymers in toluene (solid line) and decanol and benzyl alcohol (dotted line) as pore-forming diluents. PSD of polymers prepared with toluene (**a**); comparison of PSD for pDVB t and pDVB db (**b**); PSD of polymers prepared with mixture of alcohols (**c**).

Mean pore widths of the polymers prepared with toluene as the porogenic diluent are within the range of 5.0–11.6 nm, while those synthesized with the use of the mixture of the alcohols have almost twice higher values. More detailed data on porosity are provided by the pore size distribution analysis (Figure 6).

In the series of the polymers prepared with toluene as a porogenic solvent (Figure 6a), the maximum for pore size distribution is in the width range of 10–30 nm. Above this range, the pore volumes gradually decrease. The influence of pore-forming solvent(s) on the pore size distribution of pDVB is depicted in Figure 6b. The initial parts of the PSD profiles are quite similar, but above the width of 150 nm, pDVB t has very few pores, while pDVB db has a well-developed macroporous structure. This difference is a consequence of the chemical nature of solvents used as porogens. Toluene, as a thermodynamically good solvent for the polymer, promotes the formation of mesopores, while the mixture of alcohols with poorer solving properties favors the creation of macropores and, to a lesser extent, mesopores [24,25,29,30]. For this reason, in the PSD of DVB-GMA 2:1 db (Figure 6c), the main share is contributed by the macropores.

**Figure 7:**
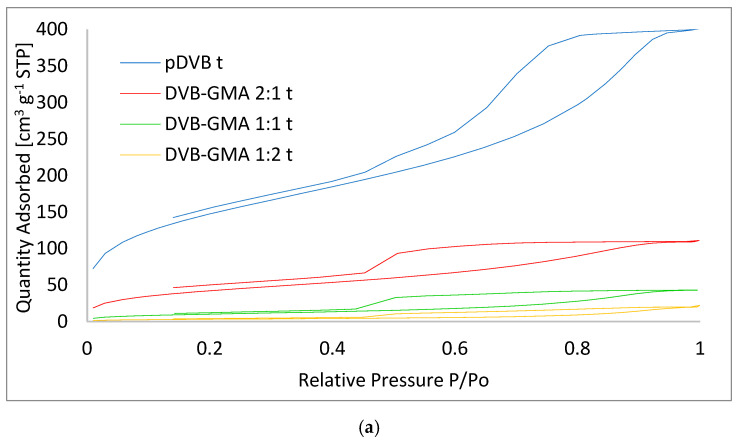

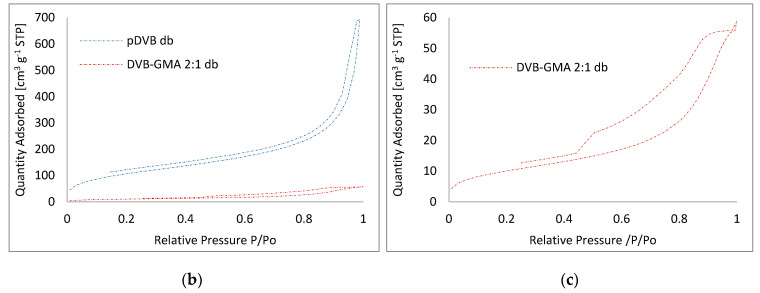
The nitrogen sorption isotherms of the studied polymers obtained in toluene (**a**) and decanol and benzyl alcohol as pore-forming diluents (**b**). Enlarged isotherm of DVB-GMA 2:1 db (**c**).

Further information regarding the porous structure of the studied materials can be found in Figure 7, where the nitrogen sorption isotherms for two different diluent systems are presented.

All the isotherms for the polymers prepared in toluene (Figure 7a) are of the same type and can be classified as IV (a). This type of isotherm is characteristic of mesoporous materials. The presence of hysteresis loops (H2(b)) indicates that capillary condensation occurs in mesopores with a width exceeding 4 nm. The shape of the loops is representative of materials with complex porous structure, in which cavities have a narrow size distribution and a neck-wide size distribution. The isotherm of pDVB db (Figure 7b) is quite different. It belongs to type V, with hysteresis resembling H3, observed for macroporous materials. In turn, DVB-GMA 2:1 db seems to combine features of both those discussed above. Its isotherm (Figure 7c) can be defined as IV(a), and the hysteresis loop resembles a combination of H2(b) and H5. Such a shape was reported for materials with irregular and complex networks of channels and cavities [31,32].

The observed differences in the shapes of the isotherms confirm the distinct mechanism of polymerization described by Gokmen and Du Prez [33] and Liu et al. [34], governed by a type of pore-forming solvent.

### 3.4. Sorption Properties

**Figure 8:**
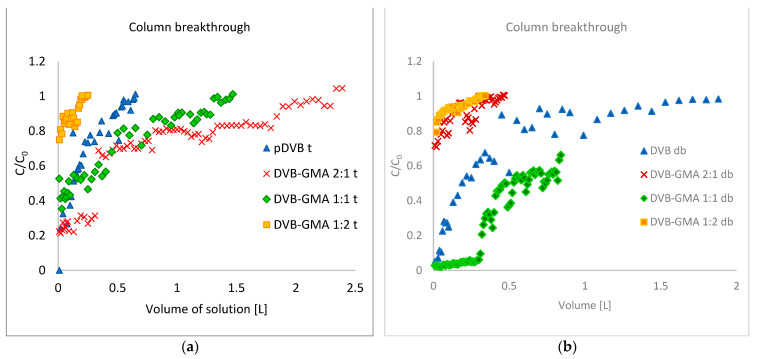
Breakthrough curves of methylene blue dynamic sorption experiments for series prepared with toluene (**a**) and with the mixture of alcohols (**b**).

Methylene blue is a model dye that is commonly used to test the sorption properties of various types of sorbents (mineral, polymeric, carbon and mixed), as it strongly interacts with solids and properly represents the chemical nature (the presence of aromatic rings and heteroatoms) of a large group of phenothiazine dyes. Additionally, its intense color makes it visible even at low concentrations [35]. Because the methylene blue molecule is large, its adsorption can be a good indicator of the mesoporous nature of the adsorbent [36]. The dynamic sorption results are presented in Figure 8, in the form of the breakthrough curves as the function of the eluate volume C/C_0_ = f (V). The interpretation of the obtained data allows us to compare the influence of the polymer chemical composition on the efficiency of methylene blue removal from aqueous solutions.

For the series of the polymers prepared with toluene, their effectiveness in water purification decreases in the following order: DVB-GMA 2:1 t > DVB-GMA 1:1 t > pDVB t> DVB-GMA 1:2 t; that for the copolymers is in agreement with their porous structure parameters, like S_BET_ and V_tot_.

Interestingly, pDVB t does not follow this trend. Although its specific surface area is the highest of all the prepared materials, it does not correlate with sorption properties. To explain this phenomenon, the nature of MB interaction with the adsorbents should be taken into account.

**Figure 9:**
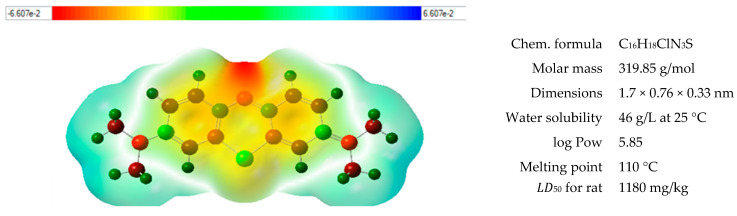
Chemical formula and chosen properties of methylene blue [38].

Methylene blue as a derivative of phenothiazinium in the form of chloride salt is a basic dye, which readily reacts with negatively charged moieties through electrostatic interactions or by cation exchange. Other possible interactions include hydrogen bonding and π-stacking [35,37]. Some data regarding MB are shown in Figure 9.

The pDVB t, being composed mainly of aromatic rings and aliphatic chains, can interact with MB only through π-π interactions. The copolymers DVB-GMA, due to the presence of the GMA monomer, gain more hydrophilic characteristics than the homopolymer. For this reason, adsorption on the copolymers is also supported by other types of interactions. It should be kept in mind that the epoxide rings of GMA are able to chemically react with MB, leading to the opening of the epoxide ring and the formation of a substitution product. In this case, adsorption is localized.

Breakthrough curves for the series prepared with a mixture of solvents (Figure 8b) do not show the same tendency as the previous one. This effect may be related to the very poorly developed porosity and broader PSD of these materials.

**Figure A2:**
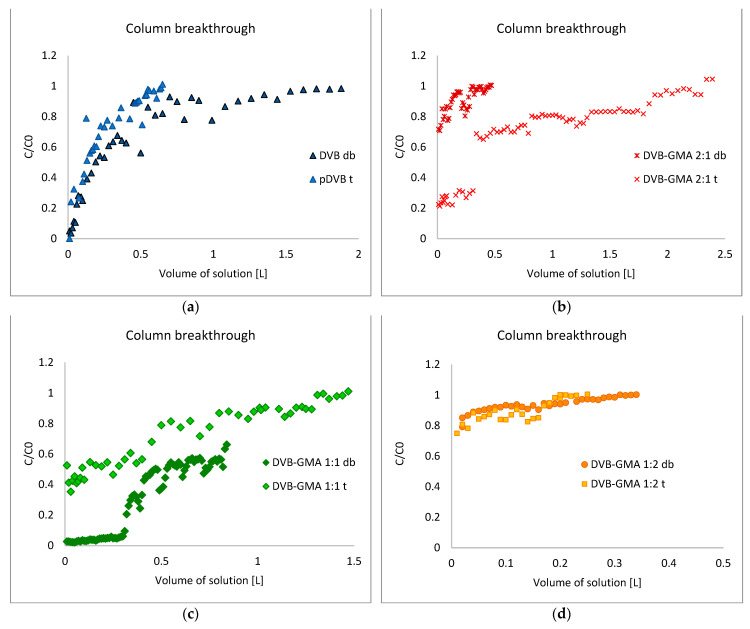
Comparison of the methylene blue sorption on the polymers, with the same chemical composition but synthesized with toluene or the decanol–benzyl alcohol mixture. The pDVB (**a**), DVB GMA2:1 (**b**), DVB-GMA 1:1 (**c**) and DVB GMA1:2 (**d**).

In order to assess the influence of pore-forming diluents on the sorption properties, a comparison of the breakthrough curves of polymers with the same chemical composition should be performed. Figure A2 presents such comparative data.

The homopolymers pDVB t and pDVB db (Figure A2a)) have very similar course of the curves, especially in the initial phase of the process. However, for samples with larger volumes, pDVB t turns out to be less effective than pDVB db. The latter material has a larger total pore volume and the pores in its structure have a wider size distribution (Figure 6b), which facilitates the transport of the large molecules of methylene blue.

DVB-GMA 2:1 t (Figure A2b) has a ca. four-fold larger S_BET_ and a ca. twice larger V_tot_ compared to DVB-GMA 2:1 db; therefore, its breakthrough occurs gradually up to a volume of 2.5 L. The much smaller active surface of DVB-GMA 2:1 db becomes saturated even at a sample volume of 0.5 L.

Although both copolymers have a low porosity, DVB-GMA 1:1 db (Figure A2c) has better sorption properties than DVB-GMA 1:1 t. The much smaller particles of the former material make the active sites (epoxy groups) on its outer surface easily accessible and, despite the inner surface not being as developed as DVB-GMA 1:1 t, MB removal is more effective.

Both DVB-GMA 1:2 materials are nonporous (Figure A2d). For this reason, they have equally poor efficiency, and no significant impact of pore-forming solvents can be observed.

A more quantitative approach to the effectiveness of the tested materials is presented in Table 6, where the quantity of methylene blue adsorbed on the polymers was designated as sorption capacity q_a_ at the point of the column breakthrough.

The polymers obtained in toluene show the highest sorption capacity for methylene blue compared to the polymers obtained in a mixture of decanol and benzyl alcohol, with the exception of the DVB-GMA 1:1 db copolymer. In the case of the materials synthesized with toluene, the highest q_a_ value is demonstrated by the DVB-GMA 2:1 t copolymer; a slightly worse value is observed for the homopolymer. The DVB-GMA 1:2 t copolymer has the worst sorption activity. These results are consistent with the data of the total pore volumes, as polymers with the highest V_tot_ values are better sorbents for methylene blue removal due to their greater porosity.

Among materials prepared with the mixture of decanol and benzyl alcohol, the DVB-GMA 1:2 db copolymer, similar to its counterpart obtained in toluene, is the worst sorbent in terms of dye sorption, while the DVB-GMA 1:1 db copolymer has the highest ability to retain methylene blue.

The desorption experiment shows that the majority of the methylene blue is adsorbed by the tested polymers irreversibly. In the case of the copolymers, a chemical reaction between methylene blue molecule and epoxy ring of GMA monomer is the main sorption mechanism. Therefore, for the DVB-GMA 1:2 copolymers, where the molar fraction of the GMA monomer was the highest, the mass of the methylene blue retained by creating an irreversible chemical bond was the highest, compared to the other tested copolymers, regardless of the type of pore-forming solution used. The chemical reaction between copolymeric adsorbents and MB elucidates the ineffectiveness of desorption processes and the high percentage of mass retained on the bed of sorbent.

Both pDVB homopolymers, despite highly developed surfaces, showed an uptake efficiency of 60–70%. As was explained above, this is a consequence of weaker interactions between MB and pDVBs. However, the MB mass values retained on these materials deserve more attention. The desorption from pDVB db with wider PSD is more effective than from pDVB t, which has less macropores in its structure. This observation lead to the conclusion that most of the large MB molecules adsorbed on pDVB t are trapped in its narrow pores, while the more spacious structure of pDVB db allows for easier removal of the adsorbates from the surface.

In order to compare the sorption capacity towards methylene blue of the studied polymers with other materials, some results of the dynamic sorption tests of MB reported in the literature are presented in Table 7.

In the literature, a variety of materials were tested as potential sorbents for the removal of MB, among them, modified silica, carbon derivatives, natural origin materials, mineral and polymeric sorbents are discussed. These materials possess specific characteristics regarding physical and chemical properties, that consequently result in different affinities towards potential adsorbates. Sorption properties are additionally affected by experimental conditions, e.g., solution concentration, the mass of sorbents, the interaction time, temperature, pH. Therefore, a reliable comparison, even with such a value as q_a_, is very demanding. In Table 7, sorption capacity is in range of 0.2–294 mg/g and recoveries 0–92 % for sorbents, from non-porous to such with very well developed porosity. The highest values of q_a_ were obtained for materials with numerous hydroxyl groups on their surface. Other materials which did not have the confirmed presence of hydroxyl groups on their surface were not so effective.

The values of the recoveries depend on the used desorption agents. The most effective was the 1 M methanolic solution of HCl, while aqueous 0.1 M HCl or NaOH and pure methanol did not exhibit a comparable effect in MB removal. Such results confirm that the mechanism of MB interactions with an adsorbent surface mainly involve chemical bonding.

Although the values of q_a_ for the polymers presented in these studies are not as high as those from the literature, the total mass of MB adsorbed on their surfaces in most cases is at the level of 70% or higher, which proves their good efficiency as adsorbents. Moreover, the amounts of MB retained on the beds are also high in all cases (57–100%), demonstrating that the dye is mainly chemically bound.

## 4. Conclusions

In this study, the influence of the pore-forming diluent and molar ratio of monomers on the structure, as well as the thermal and sorption properties of the DVB and GMA copolymers, obtained by suspension polymerization, were compared. As the pore-forming diluents, toluene and a mixture of decanol and benzyl alcohol were applied. The chemical structure of the obtained polymers was confirmed by the FTIR technique, and their chemical composition was confirmed with elemental analysis. The good agreement of the experimental and theoretical values of the CHN results proved the successful course of the syntheses. In order to determine the behavior of the tested polymers in oxidizing conditions and the course of their thermal degradation, thermogravimetric analysis was carried out both under synthetic air and a helium atmosphere. Based on the obtained results, it was found that regardless of the thermal decomposition conditions, the pore-forming diluent did not have a significant impact on the course of this process; however, the molar ratio of monomers played a significant role, as the thermal stability of the obtained polymers decreased with increasing GMA content. An analysis of the porous structure parameters allowed us to find that not only the chemical composition of the polymers but also the applied pore-forming system affected the porosity of synthetized polymers. As the DVB content increased relative to GMA, the specific surface area of the tested polymers increased. Higher values of specific surface area were observed for the systems obtained in toluene than for decanol–benzyl alcohol systems. Changes in porosity dictated by the composition of polymers and the used pore-forming solvents also translated into the sorption capacity of the tested materials towards methylene blue. Studies carried out using the dynamic sorption method showed that the polymers obtained with toluene were characterized by better abilities to remove MB from its aqueous solution than those prepared with the mixture of alcohols. The only exception was the DVB-GMA 1:1 db copolymer. The low effectiveness of desorption process was a consequence of the chemical reaction between the methylene blue molecules and the epoxy rings of GMA, leading to the irreversible bonding of dye to the surface of the polymers.

## Figures and Tables

**Table 1:** Volumes of the reagents used to the syntheses.

Polymer	DVB [mL]	GMA [mL]	t [mL]	d [mL]	b [mL]
pDVB t	30	-	45	-	-
DVB-GMA 2:1 t	20	10	45	-	-
DVB-GMA 1:1 t	15	15	45	-	-
DVB-GMA1:2 t	10	20	45	-	-
pDVB db	30	-	-	10	35
DVB-GMA 2:1 db	20	10	-	10	35
DVB-GMA 1:1 db	15	15	-	10	35
DVB-GMA 1:2 db	10	20	-	10	35

DVB—divinylbenzene, GMA—glycidyl methacrylate, t—toluene, d—decanol, b—benzyl alcohol.

**Table 2:** Assignment of the FTIR bands in the spectra of the studied polymers [20].

Pore-Forming Diluent	Copolymer	Stretching Vibrations [cm^−1^]			
		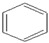	-CH_2_-	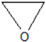	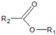
toluene	pDVB t	1596; 793; 706	2922	-	-
	DVB-GMA 2:1 t	1597; 796; 708	2926	1249; 905; 837	1726; 1187; 1110
	DVB-GMA 1:1 t	1597; 797; 709	2929	1251; 905; 840	1725; 1184; 1114
	DVB-GMA1:2 t	1597; 798; 710	2932	1249; 905; 841	1724; - ; 1122
decanol and benzyl alcohol	pDVB db	1598; 794; 707	2922	-	-
	DVB-GMA 2:1 db	1596; 796; 707	2826	1248; 903; 837	1724; 1189; 1106
	DVB-GMA 1:1 db	1596; 796; 706	2928	1248; 903; 838	1722; 1187; 1110
	DVB-GMA 1:2 db	1596; 798; 707	2929	- ; 904; 839	1720; 1183; 1119

**Table 3:** Elemental analysis with accuracy ±0.5%.

	pDVB			DVB-GMA 2:1			DVB-GMA 1:1			DVB-GMA 1:2		
	t	db	theor.	t	db	theor.	t	db	theor.	t	db	theor.
C	89.6	90.3	92.3	79.3	76.6	80.6	75.3	75.8	75.0	70.15	70.6	69.5
H	8.0	7.8	7.7	7.7	7.7	7.5	7.4	7.5	7.4	7.3	7.1	7.3
O	2.0	1.6	0	12.6	15.3	11.9	17.0	16.4	17.6	22.2	22.0	23.2

**Table 4:** The summary of the thermal analysis data of the studied polymers.

		Polymer	T_2%_ [°C]	T_range_ [°C]	T_max_ [°C]	DR * [°C·min^−1^]	Mass Loss [%]	Rm ** [%]
Atmosphere: helium	toluene	pDVB t	331.8	395–458	439.1	−14.18	82.96	6.0
		DVB-GMA 2:1 t	316.0	373–453	428.1	−11.80	87.42	4.30
		DVB-GMA 1:1 t	300.7	357–450	420.8	−9.98	88.38	4.46
		DVB-GMA 1:2 t	288.2	337–446	384.2	−8.57	90.08	4.38
	Mix of alcohols	pDVB db	333.6	393–452	431.3	−13.35	75.00	6.89
		DVB-GMA 2:1 db	307.1	365–451	423.5	−10.58	86.72	1.62
		DVB-GMA 1:1 db	279.4	361–450	418.0	−10.17	85.73	0.79
		DVB-GMA 1:2 db	311.7	360–451	418.2	−10.78	93.52	2.42
Atmosphere: air	toluene	pDVB t	309.3	299–575	329.5; 527.4	−4.34; −6.11	35.83; 62.16	4.50
		DVB-GMA 2:1 t	297.8	290–593	333.3; 528.8	−4.71; −4.57	53.69; 45.55	1.39
		DVB-GMA 1:1 t	293.2	285–564	327.9; 520.8	−4.97; −4.85	60.21; 35.87	4.12
		DVB-GMA 1:2 t	283.4	275–577	327.5; 516.8	−5.81; −4.08	68.60; 28.49	3.28
	Mix of alcohols	pDVB db	308.5	299–526	343.0; 497.8	−6.54; −9.07	44.33; 51.63	5.28
		DVB-GMA 2:1 db	303.8	303–543	342.9; 511.7	−7.86; −5.77	57.98; 39.54	2.86
		DVB-GMA 1:1 db	229.6	302–536	349.9; 507.6	−7.69; −5.39	64.92; 34.88	0
		DVB-GMA 1:2 db	301.9	307–531	351.0; 506.8	−9.20; −4.66	69.12; 27.42	3.78

* DR—decomposition rate; ** Rm—residual mass determined at 900 °C.

**Table 5:** Porous structure parameters of the studied polymers.

Pore-Forming Diluent	Polymer	S_BET_ [m^2^∙g^−1^]	V_tot_ [cm^3^∙g^−1^]	W [nm]
toluene	pDVB t	534	0.56	5.5
	DVB-GMA 2:1 t	155	0.16	5
	DVB-GMA 1:1 t	38	0.07	7
	DVB-GMA1:2 t	12	0.03	11.6
decanol and benzyl alcohol	pDVB db	396	1.05	12.3
	DVB-GMA 2:1 db	37	0.09	9.7
	DVB-GMA 1:1 db	0.2	0.002	22.1
	DVB-GMA 1:2 db	0.2	0.001	17.5

**Table 6:** The sorption capacity q_a_ and percentage adsorption and desorption efficiency.

Pore-Forming Diluent	Polymer	q_a_ [mg/g]	Total Mass Adsorbed [%]	Mass Desorbed [%]	Mass of MB Retained on the Bed [%]
toluene	pDVB t	2.02	69.43	6.53	93.47
	DVB-GMA 2:1 t	2.25	69.53	2.04	97.07
	DVB-GMA 1:1 t	1.37	72.11	4.18	95.82
	DVB-GMA 1:2 t	0.26	89.61	0.79	99.21
Decanol and benzyl alcohol	pDVB db	1.51	63.08	42.53	57.47
	DVB-GMA 2:1 db	0.39	91.71	1.16	98.84
	DVB-GMA 1:1 db	5.73	31.77	32.02	67.98
	DVB-GMA 1:2 db	0.2	94.01	0.35	99.65

**Table 7:** Efficiency of different sorbents in MB removal with dynamic method.

Adsorbents	S_BET_ [m^2^g^−1^]	C_0_ [mg/L]	q_a_ [mg/g]	Recovery [%]	Reference
Silica microspheres decorated with polydopamine	612.3	200	83.8	81 0.1 M HCl *	[39]
Hyper-cross-linked hydroxylated polystyrene (HCPS-OH 4)	69	482	293.9	92.2 1 M HCl *	[40]
Graphite oxide coated sand	Not reported	50	0.4	Not reported	[41]
Eucalyptus sheathiana bark biomass	6.55	50	30.7	46 0.1 M HCl/NaOH *	[42]
Jackfruit (Artocarpus heteropyllus) leaf powder	Not reported	100	245	Not reported	[43]
Zeolite	Not reported	30	4.5	Not reported	[44]
Rise husk	Not reported	50	6.63	Not reported	[45]
DVB-GMA 1:1 db	0–500	1	0.2–5.73	0–42 methanol *	This study

*—desorption agent.

## Data Availability

The original contributions presented in the study are included in the article, further inquiries can be directed to the corresponding authors.
